# Work safety climate. Comparison of selected occupational groups

**DOI:** 10.1371/journal.pone.0243056

**Published:** 2020-12-14

**Authors:** Marta Stasiła-Sieradzka, Agata Chudzicka-Czupała, Marta Znajmiecka-Sikora

**Affiliations:** 1 Institute of Psychology, Faculty of Social Sciences, University of Silesia in Katowice, Katowice, Poland; 2 Department of Social and Organizational Behavior Psychology, Katowice Faculty of Psychology, SWPS University of Social Sciences and Humanities, Katowice, Poland; 3 Institute of Psychology, University of Lodz, Łódz, Poland; Universitat de Valencia, SPAIN

## Abstract

Implementation of effective programs to improve occupational safety should be linked to an understanding of the specific nature of the given job. The aim of the research was to compare occupational groups with different job-related specificities: industrial production line workers, retail workers and mine rescuers, in terms of their assessment of the work safety climate. The survey covered 2,995 respondents with diversified demographic characteristics. The study used an abridged version of the Safety Climate Questionnaire by Znajmiecka-Sikora (2019) to assess 10 separate safety climate dimensions. The results of the MANOVA multivariate analysis, Wilks’ multivariate F-tests and univariate F tests prove that there is a statistically significant difference between the respondents representing the three occupational groups collectively in terms of global assessment of all work safety climate dimensions, and also indicate significant differences between workers belonging to the three occupational groups in terms of their assessment of the individual dimensions of the work safety climate, except the organization’s occupational health and safety management policy as well as technical facilities and ergonomics, which may be due to the universality of the requirements set for organizations with regard to these two aspects of safe behavior. The differences observed in the assessment of the remaining work safety climate dimensions induces one to promote more differentiated and individualized activities, taking into account the work specificity and the nature of the threats occurring in the respective working environment of the representatives of the different occupations. The difference in assessment of the work safety climate found in the research encourages one to create practical programs for safety, not only in the procedural and technical dimension, but also in the social and psychological one.

## Introduction

Researchers have recently been focusing increasingly often on the safety climate in the organization, including its significance for the way workers behave in terms of health and life protection. The employer’s task is to protect employees against threats. It often boils down to organizing safe workplaces and preventing the possibility of accidents occurring, e.g. by systematic training of workers, issuing clear instructions, making appropriate personal and collective protection equipment available, and using effective shields and safeguards on moving machine parts. However, an analysis of the research reports so far indicates that such a classic approach to understanding the employer’s role in creating a safe working environment, based only on compliance with legal requirements, is incapable of ensuring high standards of that environment [1–3]. Nor is a systemic approach to safety management, consisting in constant monitoring of its status and in implementing intervention measures on the procedural and technical level, a sufficient tool shaping a high level of safety culture within the organization, even though it does yield better results and results in lower accident rates [3–8].

Experts tend to reflect on the role of psychological mechanisms, which often play a key role in accidents [9,10]. Therefore, it is legitimate to take into account, apart from procedural and technical improvements, also the subjective feelings of workers regarding the current level of satisfaction with the safe working conditions created by their organization as well as their own attitude and subjective perception of the existing regulations and practices in this respect.

E.S. Geller [11,12] and D. Cooper [13] argue that two dimensions of safety culture need to be taken into account in its analyses. The first dimension consists in visible measures related to safety management in the organization. Studies on this aspect of safety culture rely on an audit of the procedures applied and of the workplace in terms of its safety, as well as reviews of the causes and effects of accidents that have taken place and the remedial mechanisms introduced. The organization’s security climate is the other significant, hidden dimension, related to psychological factors, behavioral patterns, values and attitudes, as well as standards adopted in the organization and their perception by the employees. Importantly, studying this dimension requires the employees to be engaged and it is impossible for researchers to use documentary analysis alone.

Similarly, R. Studencki [14], defining work safety culture as part of the overall organizational culture of a company, clearly contrasts it with the safety climate. In this author’s opinion, an organization’s safety culture refers to the state of awareness of threats, the defined standards of conduct in situations when they occur, and the technical and organizational ways to ensure the safety of employees (e.g. readiness of rescue services, restrictive monitoring of the technical efficiency of emergency alarm devices, training of employees). The safety climate is expressed by the individual beliefs of the people working in the organization regarding life and health and the need to protect them in work situations. Other researchers [15–18] define work safety climate as a dimension of the organization’s safety climate, constituting a sum of observations shared by the employees about their working environment and the organizational management system, including policy, practices and procedures aimed at ensuring safety. The work safety atmosphere reflects in a way the perceived atmosphere related to occupational health and safety. It influences workers’ attitudes and behavior in terms of health and safety at work, determining safe or risky behaviors [10,15,17,19].

It is emphasized in the literature that safety climate surveys may be considered as activities making it possible to develop practical recommendations for improving occupational safety in the organization [3,8,18,20,21]. They concern mainly spheres such as safety education, shaping standards and values, building the community and co-responsibility for others, responsible leadership, building authority, and motivating safe behaviors. Implementation of programs of modifying hazardous behaviors, based on the results of safety climate surveys, contributes to an improvement of the safety culture and of the quality of life of employees, and is also an effective tool for accident prevention. As empirical studies so far [5,15,17,20–25] have shown, studying the safety climate may constitute an important element of an appropriate and complete diagnosis of an organization’s safety culture. It is worth noting that the climate survey itself, which becomes a starting point for the implementation of practical solutions for shaping a positive safety culture of a particular organization, may be crucial for improving the safety of employees.

### Safety climate in selected occupational groups. Risk factors

Research reports [26] show that safety climate is perceived differently by staff depending on the size of the company and on the way in which work safety is managed, as well as on the gender of the employees, their seniority, their position in the organizational structure, their ethnic background, and the industry represented. Research so far points to significant differences in the evaluation of threats and in the perception of the safety climate presented by occupational groups such as underground, open-pit and borehole mines, airport ground staff, sailors, people working in polar regions, medical staff, lighting industry and construction industry workers [26–32]. Building a positive culture of work safety is therefore not marked by organizational or sectoral universality. Its appropriate creation requires identification of the factors of the work safety climate, which may constitute its important components, taking into account both the size of the organization and the way in which it is managed (including work safety management), as well as the different nature of the threats and the specificity of the tasks in different areas of occupational activity.

Planning and implementation of effective programs to improve the work safety climate should be linked to an understanding of the specific nature of the occupation and of the working conditions in the relevant organization. It is important to make sure that they take into account the assessment of the safety level, performed by the occupational group representing it operationally. The specificity of work is defined by a number of variables, including the way tasks are performed, the type and scope of hierarchical relations, customer relations, continuous contact with technology (work in a human-machine system), the level of training required for the employee and the types of threats present in the working environment. All activities aimed at developing solutions contributing to the shaping of a positive safety culture of the organization should therefore take into account the specific nature of the latter. They must not rely on the adoption of solutions implemented in other organizations without reflection. The aim of the research undertaken by the author was to continue the empirical analyses related to the comparison of the overall assessment of the work safety climate and its components, carried out by different occupational groups. The comparative groups were industrial manufacturing line workers, retail workers and mining rescuers. The choice of these occupational groups was dictated by the different nature of their work and of the threats occurring in the working environment.

Work on an industrial production line involves many potential threats whose nature is physical (e.g. moving parts of machines, noise, vibrations, electromagnetic field, high or low temperatures), chemical (toxic substances, irritants and allergens), and biological (fungi, bacteria, pathogenic viruses). Physical strain on the body may result from the need to perform work in a forced body position, repetitive activities, and excessive energy expenditure caused by strain on selected body parts. In addition, an automated working environment characterized by a routine scope of tasks and limited interactions staff members, for instance due to noise, can significantly affect the workers’ sense of threat. The work is usually carried out under in strict subordination within a hierarchical structure, paid for on an hourly or piecework basis, it is sometimes carried out in shifts, and often does not require specialized training.

Retail workers are also exposed to numerous physical risks. Their working environment often involves excessively high or low temperatures related to the storage conditions of goods (meat, dairy products), humidity (plant trade), biological hazards (in the case of trade in agricultural products and animals), and chemical hazards (trading in pesticides). The nuisance related to work in retail is caused primarily by the physical effort, related to dynamic loads (when walking) and static loads (in a standing position). In addition, retail workers are exposed to substantial psychological strain. Working in constant contact with the customer, the feeling of threat related to financial liability, the need to work during holidays, as well as intensified workloads during the so-called high season (e.g. before Christmas, during the sales period) may deepen emotional tensions, lead to greater stress and disturb the work-life balance. Work is carried out both in teams and individually, and the qualifications required from the employees tend to vary and depend on the specific range offered by the given retail outlet.

The job of a mine rescuer is a difficult and dangerous profession in which helping others in an emergency involves risking one’s own most valuable resources, life and health. This is due to the fact that rescuers have to participate in situations involving high physical strain, as the profession involves a high likelihood of injury, and mental strain, due to the frequently experienced traumatic stress. This is a highly injury-prone occupation. This is because rescuers have to participate in events causing strong physical and mental strain due to the frequent experience of traumatic stress [33–36]. The aim of the actions undertaken by rescuers is to rescue people and property at risk and to eliminate the threat posed by mining operations, caused by natural or technical factors related to mining. Rescue operations last from a dozen hours or so to several dozen days [37]. The threats associated with this occupation involve exposure to work in harmful, unpredictable conditions and rapidly changing circumstances (caving, fire, gas, and water hazards), with the possibility of various types of risk factors being activated in an unpredictable manner, with high physical effort. Workers are exposed to active participation in events during which operations are carried out under constant threat of worsening conditions, which generates and intensifies the feeling of stress. Mine rescuers always work in teams. The smallest organizational unit in mine rescue services is composed of five members in total, including four rescuers and one leader. It is a fixed group of people, whose goal is to perform activities jointly as part of the rescue operation. In mine rescue units, care is taken to make sure that the group’s composition stays permanent, its members participate together in training and rescue operations, and remain on standby duty for many days, quartered together over that time. During performance of rescue operations, the unit works in one place, and all its members must remain within sight of the others. Rescuers are required to watch one another constantly and to be attentive to the external environment, which guarantees safety on the individual and on the group level. The members of the unit operate in accordance with the “one for all, all for one” principle. Recruitment for this job involves a restrictive selection procedure. In addition to the formal requirements, training and experience related to work as a miner, the candidates are also expected to be fully mentally and physically fit and ready to participate in specialized training courses, involving improvement of the competencies required for mining rescue operations. Staying in the profession involves the requirement to undergo systematic training with regard to technical skills as well as to maintain a high level of psychophysical fitness.

### Research objectives

The study was of an exploratory nature. The aim was to compare selected occupational groups, industrial production line workers, retail workers and mine rescuers, in terms of their assessment of the work safety climate. The criterion for selection of the occupational groups was the different specificity of the work performed by their respective representatives and the different types of threats related to the environment where it is carried out. Due to the lack of research reports referring to comparative analyses with regard to the assessment of the work safety climate between the occupational groups identified in the presented studies, it was decided not to put forward hypotheses about the nature of the potential differences and similarities. The aim of the study was to fill the gap existing in this area.

The research questions put forward take into account both the differences in the assessment of the safety climate at work, considered globally, and the differences with regard to its individual components, each of its dimensions individually.

#### Question 1

Are there any statistically significant differences between workers belonging to the three occupational groups studied in terms of their global assessment of the safety climate at work safety (i.e. superposition of the distinguished dimensions in the research tool)?

#### Question 2

Are there any statistically significant differences between workers belonging to the three occupational groups studied in terms of their assessment of the individual dimensions of the work safety climate:

- employee participation in safety-related matters,
- safe behaviors,
- management engagement,
- modeling and enhancing safe behaviors in the organization,
- risk management in the workplace,
- technical facilities and ergonomics,
- pace of work and fatigue level,
- occupational health and safety training process,
- atmosphere in the workplace,
- organizational policy with regard to occupational health and safety management?

## Material and methods

The research was conducted in the territory of Poland in the period from 2017 to 2019. It was a quantitative survey carried out using questionnaires, filled out by the respondents using the paper-and-pencil method, with the written consent of the authorized representatives of the working establishments employing the respondents. All study participants gave their consent to participate in the study and received relevant information to give informed consent to participate. The research was conducted in compliance with the ethical standards in line with the provisions of the Declaration of Helsinki. The Institutional Review Board and the Ethics Committee of the Silesian University (Poland) (Reference Number KEUS 31/04/2020/ Human participants, project title: Work safety climate. Comparison of selected occupational groups) approved the research proposal and the consent procedure.

The study involved 2,995 respondents (see S1 Data. Work safety climate.xlsx). Purposive sampling was used, based on criteria related to the respondents being members of one of the three occupational groups distinguished earlier. There were 1,331 respondents working directly on production lines manufacturing various products (ceramic, lighting and food industries), i.e. 44.4% of the total sample, 1,431 respondents (47.8% of the sample) were retail workers (cosmetics, household cleaning products, food products, household appliances), while 233 respondents (7.8% of the sample) were mine rescuers (from the Central Mine Rescue Station–Centralna Stacja Ratownictwa Górniczego S.A. in Bytom, the Mine-Smelter Emergency Rescue Division of KGHM Polska Miedź S.A. in Lubań, the Mine Rescue Unit of PGNiG SA–Division of the Borehole Mining Rescue Station in Krakow). All the research results presented and the conclusions formulated on their basis refer, therefore, only to the three occupational groups presented here.

Table 1 shows the sociodemographic data of the respondents in total as a group and the data of individuals belonging to the individual occupational groups.

**Table 1:** Study sample description.

Próba	Średnia wieku	SD	Płeć	Wykształcenie	Średni staż pracy w zakładzie i zawodzie	SD
All respondents (N = 2995)	33.31	10.43	(F)1881(62.8)	(E)61(2.0%)	5.98	7.59
				(V)403(13.5)%		
			(M)1114(37.2)	(S)1908(60.7%)		
				(H)623(20.8%)		
Grupa A (Mnfc) (N = 1331)	36.44	12.05	(K)678(51%)	(P)47(3.5%)	8.15	9.45
				(Z)191(22%)		
			(M)652(49%)	(Ś)864(65%)		
				(W)129(9.5%)		
Grupa B (Retl) (N = 1431)	29.31	7.24	(K)1201(84%)	(P)14(1.0%)	2.95	2.85
				(Z)87(6.0%)		
			(M)230(16%)	(Ś)929(65%)		
				(W)401(28%)		
Grupa C (MnRsc) (N = 233)	39.83	7.19	(K)0(0%)	(Z)25(10.5%)	11.61	7.59
			(M)233(100%)	(Ś)115(49.5%)		
				(W)93(40%)		

SD–standard deviation; Gender: F-female, M-male; Education:E-elementary, V-basic vocational, S-secondary, H-higher

Group: A(Mnfc)-production workers; B(Retl)-retail workers; (MnRsc)C-mine rescuers

An abridged version of the Safety Climate Questionnaire by Znajmiecka-Sikora [38], consisting of 50 questions (see S1 Appendix. Items.pdf), was used to measure the safety climate. The tool used a Likert scale from 1 to 5, with 1 meaning “strongly disagree”, 2 –“disagree”, 3 –“hard to say”, 4 –“agree”, and 5 –“strongly agree”. Some questions require inversion.

The tool consists of 10 separate safety climate dimensions, distinguished on the basis of theoretical analysis [6,12]. The contents of the individual items in the scales was evaluated by competent judges to assess the accuracy of individual questionnaire items, and the attribution of the specific items to the individual scales and dimensions was confirmed in the course of exploratory factorial analysis. Each scale is composed of 5 questions the minimum score on each scale is 5 points and the maximum score is 25 points. Table 2 contains a brief description of each of the scales and examples of questions which they include.

**Table 2:** Description of the Safety Climate Questionnaire and contents of the individual items.

No.	Safety Climate Questionnaire scales	Description of the scale corresponding to the specific safety climate dimension *A high score on the scale means that*:	Example statements from the scale
1	Employee participation in safety-related matters	workers feel engaged by their superiors in safety-related matters and actions implemented in this field are consulted with them	I have been engaged in the process of assessment of occupational risk related to their job
2	Safe behavior	workers comply with the applicable safety procedures, use personal protective equipment and keep their working station orderly and tidy	Sometimes I engage in risky behavior at work, e.g. I remove machine guards or shields, I perform minor repairs when the machine is running, I exceed speed limits, I take shortcuts to reach my destination faster.*
3	Management engagement in health and safety	workers have a high opinion of the attitude and behavior of their superiors and their engagement in work safety aspects	My superiors “turn a blind eye” to the way in which work is done–it does not necessarily have to be done safely, the important thing is to meet the deadlines and that the quantity is appropriate.*
4	Modeling and enhancing safe behaviors in the organization	safe way of performing tasks, compliance with safety rules is noticed and appreciated by superiors, and their attitude towards safety is taken into account in the employee performance appraisal process	My superiors actively participate in campaigns promoting safety organized by the company.
5	Workplace accident risk management	workers rate positively the accident prevention and response measures undertaken by the organization	Each accident that has occurred at the working establishment is discussed by the superior during information meetings.
6	Technical facilities and ergonomics	workers have a high opinion of both the technical condition of the plant and machinery and the way in which work is organized	Particularly dangerous places are suitably marked.
7	Pace of work and fatigue level	workers rate positively the way work is organized and do not feel overtired	The number of tasks I have to perform every day makes me work at a very fast pace.*
8	Occupational health and safety training process	workers feel well informed about aspects related to safety, occupational risk, job-related threats, and methods of providing first aid	I feel well informed about the methods of protecting myself from the threats related to my work.
9	Atmosphere in the workplace	workers rate positively the communication method and the level of support provided by the superior, and trust one another	When dangerous work is performed, I know I can trust my co-workers with whom I perform the dangerous task.
10	Organizational policy with regard to occupational health and safety management	workers recognize and rate positively the actions undertaken by the employer to improve safety in the organization	Safety is a priority in our organization, and a continuous improvement process with regard to safety is in course.

*Question requiring inversion

Source: compilation based on Znajmiecka-Sikora [38].

The individual results show how specific workers perceive the work safety climate in the organization employing them in the individual dimensions. The overall result, obtained by all the employees, reflects the work safety climate in the organization, and makes it possible to conclude on the level of safety culture in the company. It also allows comparisons to be made this respect with results obtained in other organizations and occupational sectors.

Reliability of the entire Safety Climate Questionnaire, calculated using Cronbach’s alpha, is 0.93, ranging for the individual scales of the questionnaire between 0.68 and 0.89 [38], and for the presented study from 0.61 to 0.88. Table 3 indicates the Cronbach’s alpha values for the individual scales in the tested samples.

**Table 3:** Scale reliability: Cronbach’s alpha for the individual scales of the Safety Climate Questionnaire in the individual respondent groups.

Safety Climate Questionnaire scales	Group A(Mnfc)	Group B(Retl)	Group C(MnRsc)
Employee participation in safety-related matters	α = 0.67	α = 0.61	α = 0.77
Safe behavior	α = 0.70	α = 0.70	α = 0.75
Management engagement in health and safety	α = 0.71	α = 0.75	α = 0.83
Modeling and enhancing safe behaviors in the organization	α = 0.77	α = 0.74	α = 0.81
Workplace accident risk management	α = 0.76	α = 0.67	α = 0.81
Technical facilities and ergonomics	α = 0.72	α = 0.73	α = 0.84
Pace of work and fatigue level	α = 0.77	α = 0.81	α = 0.77
Occupational health and safety training process	α = 0.69	α = 0.74	α = 0.88
Atmosphere in the workplace	α = 0.73	α = 0.78	α = 0.79
Organizational policy with regard to occupational health and safety management	α = 0.78	α = 0.77	α = 0.85

### Statistical data analysis methods

In the first stage of the analysis, descriptive statistics of the variables and correlation coefficients between the results of the individual Safety Climate Questionnaire scales were calculated, separately for the individual respondent groups (representatives of the three occupational groups distinguished).

A MANOVA multivariate analysis was conducted to find answers to the research questions put forward (cf. Table 6). Multivariate Wilks’ F tests were used to find an answer to question 1, concerning the existence of a statistically significant difference between the representatives of the three occupational groups examined together and the three groups of workers compared in pairs (production workers to retail workers, production workers to mine rescuers, and retail workers to mine rescuers) in terms of the global assessment of the safety climate at work (superposition of the assessment of all safety climate dimensions). Univariate F tests were used to find an answer to question 2, concerning the existence of differences in the assessment of the individual work safety climate dimensions, made by representatives of all three occupational groups together, and then for the occupational groups analyzed in pairs.

The adoption of such an analysis model is “an optimal choice when the researcher treats the explained measurable variables as interrelated and thus as ones creating a relational structure of properties which can be meaningfully and substantively interpreted, in whole or in part” [39], and this is the case with respect to the assessment of the individual dimensions of the work safety climate. All statistical analyses were carried out using the STATISTICA 13 statistical package.

## Results

Descriptive statistics concerning all the studied variables (the mean values obtained and the standard deviations) for each group of respondents, representing the three occupational groups, are presented in Table 4.

**Table 4:** Descriptive statistics of the studied variables.

Variables	Group A (Mnfc) (N = 1,331)		Group B (Retl) (N = 1,431)		Group C (MnRsc) (N = 233)	
	M	SD	M	SD	M	SD
Employee participation in safety-related matters	18.59	3.08	18.07	2.97	18.61	3.83
Safe behavior	19.93	3.51	20.75	2.94	18.34	3.81
Management engagement in health and safety	20.12	3.43	21.32	2.90	18.64	4.13
Modeling and enhancing safe behaviors in the organization	18.06	3.05	18.59	2.85	17.64	3.43
Workplace accident risk management	18.99	3.17	16.94	3.16	19.56	3.20
Technical facilities and ergonomics	20.56	2.38	20.35	2.53	20.51	2.82
Pace of work and fatigue level	13.18	3.82	14.10	4.24	16.52	3.59
Occupational health and safety training process	19.49	2.74	20.36	2.38	20.81	2.76
Atmosphere in the workplace	19.27	2.68	20.62	2.83	20.17	2.84
Organizational policy with regard to occupational health and safety management	19.23	2.66	19.21	2.67	19.28	3.19

M–mean, SD–standard deviation Sample:A(Mnfc)–production workers; B(Retl)–retail workers; C(MnRsc–mine rescuers

Table 5 contains the intercorrelations between the studied variables separately for the three occupational groups studied. All variables are significantly correlated with a significance level of p = 0.01. The correlation in the vast majority of the cases is weak or average, and only single coefficients reach 0.7, indicating a high correlation.

**Table 5:** Correlations between the studied variables.

Variable	1	2	3	4	5	6	7	8	9
**Group A** (Mnfc)–**Production workers (N = 1,331)**									
1. Employee participation in safety-related matters									
2. Safe behavior	0.17								
3. Management engagement in health and safety	0.30	0.55							
4. Modeling and enhancing safe behaviors in the organization	0.48	0.36	0.50						
5. Workplace accident risk management	0.49	0.28	0.37	0.51					
6. Technical facilities and ergonomics	0.43	0.37	0.50	0.49	0.47				
7. Pace of work and fatigue level	0.17	0.19	0.30	0.28	0.09	0.22			
8. Occupational health and safety training process	0.41	0.29	0.40	0.48	0.44	0.52	0.19		
9. Atmosphere in the workplace	0.29	0.30	0.42	0.45	0.31	0.45	0.30	0.48	
10. Organizational policy with regard to occupational health and safety management	0.56	0.35	0.52	0.59	0.61	0.59	0.26	0.53	0.48
**Group B**(Retl)–**Retail workers (N = 1,431)**									
1. Employee participation in safety-related matters									
2. Safe behavior	0.28								
3. Management engagement in health and safety	0.28	0.60							
4. Modeling and enhancing safe behaviors in the organization	0.49	0.40	0.52						
5. Workplace accident risk management	0.41	0.21	0.25	0.47					
6. Technical facilities and ergonomics	0.36	0.34	0.39	0.41	0.49				
7. Pace of work and fatigue level	0.28	0.40	0.39	0.39	0.20	0.19			
8. Occupational health and safety training process	0.43	0.36	0.42	0.48	0.36	0.52	0.22		
9. Atmosphere in the workplace	0.32	0.41	0.51	0.47	0.19	0.34	0.37	0.53	
10. Organizational policy with regard to occupational health and safety management	0.44	0.40	0.50	0.58	0.52	0.56	0.31	0.55	0.45
**Group C**(MnRsc)**–Mine rescuers (N = 233)**									
1. Employee participation in safety-related matters									
2. Safe behavior	0.44								
3. Management engagement in health and safety	0.48	0.74							
4. Modeling and enhancing safe behaviors in the organization	0.64	0.52	0.64						
5. Workplace accident risk management	0.53	0.30	0.42	0.61					
6. Technical facilities and ergonomics	0.62	0.53	0.61	0.68	0.58				
7. Pace of work and fatigue level	0.19	0.54	0.55	0.41	0.19	0.40			
8. Occupational health and safety training process	0.56	0.48	0.54	0.60	0.67	0.73	0.28		
9. Atmosphere in the workplace	0.48	0.47	0.58	0.65	0.61	0.65	0.39	0.76	
10. Organizational policy with regard to occupational health and safety management	0.62	0.57	0.67	0.71	0.68	0.75	0.42	0.72	0.68

p = 0.01

The results shown in Table 6 were analyzed to answer question 1: they prove the existence of a statistically significant difference between the studied representatives of the three selected occupational groups together in terms of the global assessment of all work safety climate dimensions. The multivariate Wilks’ F test value (F = 69.18, p = 0.00), indicates that all the three groups differ significantly in this respect. The results (cf. Table 6) also make it possible to conclude that in the global assessment of the work safety climate, there are significant differences between representatives of all the occupational groups analyzed in pairs: industrial production line workers with retail workers (F = 82.14, p = 0.00), industrial production line workers with mine rescuers (F = 42.46, p = 0.00) and retail workers with mine rescuers (F = 72.00, p = 0.00).

**Table 6:** Results of MANOVA variance analysis (multivariate and univariate tests).

All effects (Wilks multivariate test): F = 69.18p = 0.00 partial eta-squared = 0.19								
Variables	Univariate test for 3 groups A, B, C		Multivariate test (for groups A and B) F = 82.14 p = 0.00 eta-squared, partial = 0.23		Multivariate test (for groups A-C) F = 42.46 p = 0.00 eta-squared, partial = 0.21		Multivariate test (for groups B-C) F = 72.00p = 0.00 eta-squared, partial = 0.30	
			Univariate test for groups A-B		Univariate test for groups A-C		Univariate test for groups B-C	
	F	Adjusted R^2^	F	Adj. R^2^	F	Adj. R^2^	F	Adj. R^2^
1. EPSRM	10.97**	0.01	20.96**	0.01	0.01	-0.00	6.21*	0.00
2. SB	62.68**	0.04	45.45**	0.02	39.42**	0.02	123.39**	0.07
3. MEH&S	91.69**	0.06	99.00**	0.03	34.47**	0.02	149.11**	0.08
4. M&ESBO	16.68**	0.01	22.55**	0.01	3.49	0.00	20.77**	0.01
5. WARM	173.12**	0.10	289.70**	0.09	6.35*	0.00	137.52**	0.08
6. TF&E	2.46	0.00	4.93*	0.00	0.09	-0.00	0.74	-0.00
7. PWFL	72.74**	0.05	35.80**	0.01	153.39**	0.09	67.69**	0.04
8. OH&STP	52.07**	0.03	80.88**	0.03	45.97**	0.03	6.61**	0.00
9. WA	81.90**	0.05	163.46**	0.06	21.66**	0.01	5.04*	0.00
10. OOH&SMP	0.09	-0.00	0.04	-0.00	0.08	-0.00	0.16	-0.00

significance of differences between mean values for study samples A, B and C

** p = 0.01

* p = 0.05

Group: A(Mnfc) (N = 1,331)–production workers; B(Retl) (N = 1,431)–retail workers; C (MnRsc) (N = 233)–mine rescuers

Variables:

EPSRM–Employee participation in safety-related matters

SB–Safe behavior

MEH&S–Management engagement in health and safety

M&ESBO–Modeling and enhancing safe behaviors in the organization

WARM–Workplace accident risk management

TF&E–Technical facilities and ergonomics

PWFL–Pace of work and fatigue level

OH&STP–Occupational health and safety training process

WA–Atmosphere in the workplace

OOH&SMP–Organizational policy with regard to occupational health and safety management

Univariate F tests were performed to find an answer to question 2. Calculations were performed for the assessments of the individual work safety climate dimensions for the three occupational groups together and for all the groups compared in pairs. The results (cf. Table 6) suggest that all the studied worker groups together differ significantly in terms of the following dimensions: employee participation in safety matters, safe behaviors, management’s commitment to health and safety, modeling and strengthening safe behaviors in the organization, workplace accident risk management, pace of work and level of fatigue, health and safety training process, and atmosphere in the workplace. For the areas mentioned above, the values of the univariate F test are statistically significant. However, no statistically significant differences were found between the three occupational groups studied together with regard to the assessment of the other work safety climate dimensions, such as technical facilities and ergonomics, as well as the organizational policy with regard to occupational health and safety management.

The analysis of the differences between the assessment made by representatives of the individual occupational groups analyzed in pairs proves the existence of statistically significant differences between all the groups studied. Significant differences occur between industrial production line workers and retail workers in all the work safety climate dimensions studied, except the organizational occupational health and safety management policy dimension. Similarly significant differences were found between industrial production line workers and mine rescuers. These are related to the assessment of the following work safety climate dimensions: pace of work and fatigue level, occupational health and safety training process, safe behavior, management engagement in occupational health and safety, workplace atmosphere and workplace accident risk management. Industrial production line workers and mine rescuers do not differ significantly in terms of their assessment of the following four work safety climate dimensions: employee participation in safety-related matters, modeling and enhancing safe behaviors in the organization, technical facilities and ergonomics, and organizational occupational health and safety management policy. The results also prove the existence of statistically significant differences between retail workers and mine rescuers in terms of their assessment of eight out of ten safety climate dimensions. No significant differences were found here only in the assessment of climate aspects such as technical facilities and ergonomics, and the organizational policy with regard to occupational health and safety management.

### Summary of the research findings

The results prove the existence of significant differences between workers belonging to the three occupational groups studied in terms of their assessment of the work safety climate in the organization employing them. The differences found between the representatives of all three occupational groups together concern the global assessment of all work safety climate dimensions. The effect size (cf. Table 6) is relatively high here, as 19% of the variance of the global safety climate assessment is explained by the determining factor, i.e. the fact of belonging to the specific occupational group.

The results also confirm the existence of significant differences in the global assessment of all work safety climate dimensions between industrial production lines workers and retail workers (23% of the variance of the global work safety climate assessment is explained here by the factor of belonging to the occupational group), between industrial production lines workers and mine rescuers (21% of the variance of the global work safety climate assessment is explained by the factor of belonging to the occupational group) and between retail workers and mine rescuers (where belonging to the occupational group explains as much as 30% of the variance of the global work safety climate assessment). Question 1 can be answered affirmatively.

The univariate F test values and effect sizes measured using adjusted R^2^ (cf. Table 6) indicate that all the studied worker groups together differ significantly between one another in terms of the assessment of most work safety climate dimensions, and particularly strongly in their assessment of the following dimensions: workplace accident risk management (the lowest rating was given to this dimension by retail workers, and the highest by mine rescuers), management engagement in occupational health and safety (rated lowest by mine rescuers, and highest by retail workers), workplace atmosphere (rated lowest by industrial production line workers, and highest by retail workers), pace of work and fatigue level (rated lowest by industrial production line workers, slightly higher by retail workers, and highest by mine rescuers), safe behavior (this safety culture aspect was rated lowest by mine rescuers, and highest by retail workers) and the occupational health and safety training process (rated lowest by industrial production line workers, and highest by mine rescuers).

All three occupational groups together also differed significantly between one another in terms of the assessment of the following dimensions: modeling and enhancing safe behaviors in the organization (this safety culture aspect was rated lowest by mine rescuers, and highest by retail workers) and employee participation in safety-related matters (rated lowest by retail workers and highest by mine rescuers). There are no significant differences between the studied representatives of the three occupational groups together in terms of their assessment of the organizational occupational health and safety management policy and the technical facilities and ergonomics dimensions. It can be concluded on the basis of the results that questions 2a, 2b, 2c, 2d, 2e, 2g, 2h and 2i can be answered affirmatively, while questions 2f and 2j should be answered negatively.

The results also indicate the existence of a significant difference between the assessments given by the respondents when the analyses involved comparisons of two occupational groups each time (univariate F test values and effect sizes measured with adjusted R^2^, see Table 6). Significant differences were found between production workers and retail workers in most of the studied work safety climate dimensions, except the organizational occupational health and safety management policy dimension. These differences concern mainly the following dimensions: workplace accident risk management (rated much higher by production workers), workplace atmosphere, management engagement in health and safety and occupational health and safety training process (the last three dimensions rated significantly higher by retail workers).

Similarly significant differences between industrial production line workers and mine rescuers in terms of their judgments concern six dimensions of work safety climate. The main differences between these two groups concern their assessments of work safety climate aspects such as the pace of work and fatigue levels and the occupational health and safety training process (these dimensions are rated much higher by mine rescuers). Significant differences also concern the assessment of the following work safety climate dimensions: safe behavior and management engagement in health and safety (dimensions rated much higher by production workers) as well as workplace atmosphere and workplace accident risk management (rated higher by mine rescuers). Industrial production line workers and mine rescuers do not differ in terms of their assessment of employee participation in safety-related matters, modeling and enhancing safe behaviors in the organization, technical facilities and ergonomics, and organizational occupational health and safety management policy.

The results also prove the existence of statistically significant differences in the assessment of most dimensions of the work safety climate between retail workers and mine rescuers. They concern mainly aspects such as management engagement in health and safety and safe behavior (rated significantly higher by retail workers), workplace accident risk management, and pace of work and fatigue levels (rated significantly higher by mine rescuers). Significant differences between these occupational groups were also observed in the assessment of the following dimensions: modeling and enhancing safe behaviors in the organization and atmosphere in the workplace (rated significantly higher by retail workers), as well as the occupational health and safety training process and employee participation in safety-related matters (perceived more positively by mine rescuers). No significant differences were found between retail workers and mine rescuers in terms of their assessments of work safety climate aspects such as technical facilities and ergonomics, and the organizational policy with regard to occupational health and safety management.

## Discussion

The lack of differences observed in the presented results between the studied groups in their assessment of such work safety climate dimensions as organizational occupational health and safety management policy and technical facilities and ergonomics may result from the fact that in practice, they constitute the basic and often the only area of activities in many organizations. Initiatives of this nature are highly visible to the employees, as they are related to the reward and penalty systems in place in the respective companies, as well as systematic training and instructions related to the safe use of tools and behavior in the workplace, whose nature is universal [5,40].

With regard to the differences observed between the groups, it was noted that industrial production line workers did not rate any of the studied work safety climate dimensions significantly higher than representatives of the other groups. On the other hand, they rated significantly lower dimensions such as pace of work and fatigue level, occupational health and safety training process, and atmosphere in the workplace.

The differences observed are justified by the specific nature of the work performed by members of this occupational group. Their work involves physical strain, which is certainly linked to the level of fatigue. Limited contact with other colleagues, due to the specificity of the place of work (single-workstation job) and the competitive reward system, may have a significant impact on the lower rating attributed to social relations. The process of training or informing employees about safety procedures is different among production workers compared to the other occupational groups analyzed. Information meetings are organized for the whole staff (shift), and training is usually held for large groups, which may affect both the quality of the communication and the effectiveness of the training as such [41].

Compared to the other occupational groups, retail workers declare the safest behaviors, giving the highest rating to the management’s engagement in health and safety, modeling and enhancing safe behaviors, and the workplace atmosphere. They also give the lowest rating, compared to the other groups, to the process of participation in safety-related matters and accident risk management. Compared to production workers, retail workers declare a lower level of fatigue and a slower pace of work, and they give a better rating to the occupational health and safety training process. The situation is totally different when retail workers are compared to mine rescuers. Retail workers declare a higher level of fatigue than mine rescuers, as well as a faster pace of work, and they give a lower rating to the training process.

Also in this case, the results obtained are justified by the specific nature of the work performed by the study respondents. Work in retail usually does not require one to use personal protective equipment, with a lower level of psychophysical strain than in the case of mine rescuers and production workers. Their work is carried out mostly in small teams, which makes it undoubtedly easier to build interpersonal relationships and an appropriate atmosphere. Training with regard to safety rules is held most often in small groups, which improves the effectiveness of such processes [41] The possibility of having direct contact with one’s superior makes it possible to model appropriate attitudes towards safety rules in this case.

Mine rescuers, compared to representatives of the other two occupational groups, rate significantly higher such work safety climate dimensions as pace of work and fatigue levels, occupational health and safety training process, and organizational policy with regard to health and safety management. In addition, representatives of this occupation group rated significantly higher the atmosphere in the workplace compared to production line workers, and employee engagement in safety-related matters compared to retail workers. They gave a significantly lower rating, on the other hand, to work safety climate dimensions such as safe behavior and management engagement. Compared to retail workers, they also rated significantly lower modeling and enhancement of safe behaviors in the organization as well as atmosphere in the workplace.

The results obtained are justified by the specific nature of work in mine rescue services. Referring to the differences noted between the groups in their assessment of the pace of work and fatigue level, it is worth pointing out that more positive assessment in the rescuer group may be explained by the fact that, although the conditions in which rescue operations are carried out involve a huge physical and mental strain, the circumstances of extreme strain are currently treated as extraordinary. Representatives of the other two groups, in turn, are often exposed to long-term stimulation, accompanied by high expectations regarding quality and performance standards, often constituting a regulator of production or sales profitability [42], and this often results in a high pace of work and in high fatigue accompanying it. Compared to them, a significant part of rescuers’ working time is taken up by long periods of relative calm, waiting, and being on alert. The psychophysical strain during drills is not as high as the level of stimulation during a real rescue operation either [37]. Therefore, the work of mine rescuers involves operating in extreme conditions, from minimum to maximum stimulation, from long periods of wait, training preparing for activities and drills to stay physically fit to actual participation in emergency situations during the rescue operation. All routine activities performed by rescuers are also subject to appropriate planning, and much care is taken to make sure their work is healthy, including ensuring an appropriate balance between activity and rest time. Also, as rescuers are required to stay at the rescue stations, this forces them to function according to a suitable daily rhythm.

Significantly higher ratings attributed to such work safety climate dimensions as occupational health and safety training and workplace accident risk management by mine rescuers may also be associated with the specific nature of their work. Mine rescuers, representing a difficult and dangerous profession, experience negative effects resulting from the threats associated with the occupational activities undertaken, both at the somatic and at the psychological level. Their minimization is based on a good knowledge of the ways of coping in an emergency situation at the cognitive level (recognition and appropriate assessment of the threat and of the related risk) and at the technical level (appropriate use of measuring and protective equipment), physical fitness and mental resilience, as well as proper communication and coordination of work within the rescue unit. This process is based on continuous training of the rescuers, based on the analysis of past situations and on simulation of behavior in potential situations that might occur in the future [35].

The scope of responsibility of superiors in this particular occupational group is also not without importance for the explanation of the significantly lower rating attributed by mine rescuers, compared to industrial production line workers, to such dimensions of the work safety climate as safe behaviors and management engagement. The composition of the rescue unit is not random. Care is taken to make sure that it is permanent, that the unit is composed of suitably selected people who complete drills together before proceeding to a rescue operation. During rescue actions, the rescuer must absolutely obey the orders of the unit leader, and is not allowed to comply with third-party orders. Sometimes it is impossible to contact higher-level superiors during the action, and the conditions may be extremely dangerous. In such a situation, the unit operates as if it were autonomous, with decisions made only by the unit leader, who bears individual responsibility for them [35]. Industrial production line workers, in turn, are subject to constant, direct supervision during the performance of their work, with the aim of eliminating on a systematic basis any behaviors and situations posing a threat. The composition of worker groups is frequently modified depending on the shift work rhythm or recruitment of new team members. The activities of managers and representatives of occupational health and safety services are watched by employees in everyday practice and focus on modeling behaviors in line with the procedures in force and with the job training given to new hires. Workers are expected rather to contribute to the creation of a safe working environment than to make decisions autonomously when a threat occurs [5,6,41].

Compared to retail workers, mine rescuers also rated significantly lower the dimensions involving modeling and enhancing safe behaviors in the organization and atmosphere in the workplace. The results obtained confirm the results of previous research in this area [42–44], related to the importance of the sense of mission and bonding within the rescuer group. They show that although these aspects are important, appropriate equipment, training and effective management are more significant determinants of the feeling of safety and minimization of the stress perceived by mine rescuers. These aspects become particularly important in situations where people risk their lives or health.

An examination of the safety climate in an organization constitutes an invaluable source of knowledge not only for theoreticians who describe its characteristics and search for premises significant for an adequate diagnosis, but also for employers, health and safety employees, and work psychologists. It has been demonstrated many times that the level of safety culture is significantly related to the safety climate and translates directly both into the number of risky behaviors undertaken and the accident rate in the organization. Consequently, all activities contributing to the identification of the factors influencing the safety climate experienced subjectively by the workers can be of huge importance. The next step, after capturing the subjective perception of the safety-related factors in the workplace, should consist in designing of specific prevention strategies on the basis of this knowledge. As a result, this can help to reduce the number of accidents. Consequently, such measures should be given the highest priority, especially in occupations where people are at risk of losing their lives.

The presented results of the study of the differences in the perception of the work safety climate by the occupational groups distinguished make one inclined to take into account, both in the diagnosis and during the formulation of practical recommendations, of such dimensions of work as the nature of the threats and the possible consequences resulting from failure to comply with safety rules (in terms of economic losses and damage to health). Important factors are include the scope and direct nature of management supervision, including the role of managers in modeling safe behaviors among the staff, co-responsibility for their own and their co-workers’ safety, and the manner of conducting the required training to shape safe behaviors in the workplace and raise awareness of the existing risks.

A diagnosis of the work safety climate, taking into account the specificities of the individual sectors, makes it possible to provide recommendations containing guidelines on measures contributing to the shaping of a high culture of work safety and of the safety of the people employed in the organization. Recommendations, depending on the outcome of the diagnosis, may concern both training and other possible forms of impact, such as e.g. shaping the appropriate attitudes and making sure that appropriate role models are provided, which is in particular the role of leaders and managers, as well as the scope of implementation of motivation programs, implemented by the departments dealing with safety and HRM. Emphasis should be placed on the appropriate manner of communicating important recommendations to the subordinates, acting on these recommendations within the teams, and modifications related to work ergonomics to reduce physical effort and excessive mental strain (monitoring workers’ health and well-being).

### Research limitations

The limitations of the research include reducing the results obtained to only three occupational groups. It is advisable that other groups be taken into account in further work, exposed to threats of a different nature in the workplace. The limitations of the research also include the unequal proportion of women and men in the occupational groups studied, which may have determined the results of the study. In the research presented here, there was a more or less equal balance between men (49%) and women (51%) in the industrial production line worker group, while the group of retail workers was dominated by women (84% of the respondents), and the mine rescuer group included only men due to the legal restrictions excluding women from this profession. As it has already been demonstrated [45], gender is an important factor that may differentiate the perception of the work safety climate. The research results indicate that women differ from men in terms of the types of threats identified, they may react differently to stress, and assess individual dimensions of the work safety climate differently from men [46].

An important aspect may also be the position of the studied individuals within the organizational structure. Research [47] proves that the higher this position is, and consequently the more responsible the respective individuals are for shaping the safety culture in the organization and the larger impact they have on it, the more positively they rate the safety climate. This variable was not taken into account in the studied groups.

Schwatka et al. [26] and Noort et al. [48] also point to the differences in the perception of the work safety climate resulting from the form of employment (e.g. workers employed by construction subcontractor companies rate climate lower). The research presented in this paper does not take into account data such as the legal form of the employment contract either. It is possible that the survey respondents employed in industry or retail included temporary employment agency workers.

### Directions for future research

We believe that the results obtained may provide the basis for the development of programs aimed at modifying dangerous behaviors, based on studies of multiple dimensions of the work safety climate, which can be implemented in practice. Such programs, for instance in the form of workshops, could contribute to an improvement of the safety culture and of the quality of life of workers representing the occupational groups distinguished.

The research presented in this paper did not take into account individual factors related to personality traits or other inherent predispositions of individuals working in the relevant occupations, either. We believe that it would be desirable to supplement the research model with external and organizational determinants as well as ones related to the specific nature of the work performed by representatives of the individual occupational groups, physical and mental health, as well as selected psychological traits of the employees, such as personality and temperamental characteristics, e.g. perseverance, conscientiousness, motivation of achievements, willingness to compete, sense of coherence and self-efficacy, dispositional optimism and ways of coping with stress, social support and other traits, which may translate into the way in which people behave in the workplace in safety-related situations [49]. National culture may also constitute an important variable, so comparisons between organizations with similar work specificities, operating in different countries and on different continents, could lead to some interesting insights. Combining these factors into a single coherent model would make it possible to provide a fuller picture of the significant determinants of work safety climate assessment determinants. This could be achieved by using a cultural adaptation of the tool used in the presented research to study the safety climate in the organization (version in English and Polish in the appendix), potentially applied in broader analyses of the determinants of safe behavior of employees.

The results of systematic research could also serve as a basis for comparing individual units within the organization, between branches of the organization located in different countries, or workplaces with a similar activity profile. This in turn would make it possible to identify the most effective operating practices within the specific company as well as among the entities operating in the analyzed industry. Research on the work safety climate in individual organizations could therefore constitute an element of benchmarking in the management of safety in the organization and in the industry as a whole.

## Conclusions

The research results obtained are in line with the previous reports of other researchers, presenting differences in the assessment of the work safety climate by representatives of different occupational groups. No differences were found in the present research between the studied representatives of the three occupational groups together only with regard to work safety climate dimensions such as organizational occupational health and safety management policy and technical facilities and ergonomics. This may result from the universality of the requirements, present in the law, set for organizations precisely with regard to these aspects of safe behavior. However, the differences observed in the assessment of the remaining work safety climate dimensions organizations induce the promotion of additional activities in organizations in a more differentiated and individualized way, which should take into account the specificity of the work and the nature of the specific threats occurring in the working environment of the representatives of the different occupations.

Learning about the safety climate and diagnosing it has not only theoretical, but also applicational significance. The difference revealed in the assessment of the work safety climate thus becomes a reason for working on practical safety measures, not only in the procedural and technical dimension, but also taking into account its other aspects, including social and psychological ones. It can be the basis for creating and implementing prevention program, designed adequately to the diagnosed specificity of the issues, and for strengthening the regulations and practices functioning in organizations that require corrective actions.

## Supporting information

S1 Data Work safety climate. (XLSX)

S1 Appendix Items. (PDF)
