# Supplementary material for: Work safety climate. Comparison of selected occupational groups
Source: PLoS One. 2020 Dec 14;15(12):e0243056. doi: 10.1371/journal.pone.0243056 (PMC7735595; doi:10.1371/journal.pone.0243056)
Supplement: S1 Appendix — (PDF) [file pone.0243056.s002.pdf]

Safety Climate Questionnaire (KKB) – short version

Advice:

This questionnaire contains statements which present human behaviour in various occupational situations and work institutions. Read each statement carefully and circle the answers to show the extent to which the given statement is true for you (based on your work experience).

*Circle **DY** – if you want to answer **definitely yes**; Y – if you want to answer **yes**, **DN** – if you want to answer - **don't know**; **N** – **no**, DN – **definitely no**. Please answer as if your objective were to give a reliable description of your behaviour at work, the behaviour of your superiors, and work organisation.*

No.	*question*	*replies:*<br>*DY – definitely yes*<br>*Y – yes*<br>*DK – don't know*<br>*N – no*<br>*DN – definitely no*
1.	The machines and devices I operate undergo interim check-ups.	DY Y DK N DN
2.	I have been involved in the occupational risk assessment process at my workstation.	DY Y DK N DN
3.	The employer's actions linked to occupational safety are consulted with employees.	DY Y DK N DN
4.	From time to time, I happen to perform my duties not in compliance with the rules of occupational safety and health.	DY Y DK N DN
5.	Employees can test personal protection equipment (*e.g. footwear, goggles and glasses*) prior to the decision to purchase them.	DY Y DK N DN
6.	In our firm employees are members of the teams working to improve safety within the organisation (*e.g. they participate in formulating safety procedures, OHS instructions, in the works of post-accident teams, risk assessment, OSH committee*).	DY Y DK N DN
7.	My workstation is orderly and tidy (tools are kept in the same place and waste is removed on a current basis) and it is important for me to keep the workstation tidy.	DY Y DK N DN
8.	Employees are advised of any implementation of long-term safety projects (*e.g. ISO, OHSAS, prophylactic programmes, behaviours at the workstation monitoring programme*).	DY Y DK N DN
9.	My employer takes measures to improve safety within the organisation.	DY Y DK N DN
10.	My superiors are not interested in OSH issues.	DY Y DK N DN
11.	From time to time my superior happens to assign a task to be fulfilled contrary to the rules of occupational safety (*e.g. to remove the protective shield from the machine so that it works faster*).	DY Y DK N DN
12.	My superior intervenes whenever the work safety rules are being breached.	DY Y DK N DN
13.	I disregard my superior's remarks and instructions on safety (*e.g. I do not wear protective gloves, footwear, hearing protectors, even though my superior has admonished me to wear them*).	DY Y DK N DN
14.	From time to time, I happen to behave riskily (*e.g. I remove protective shields from machines, carry out minor repairs while the machine is in operation, exceed the allowable speed, take short-cuts to hit the target more quickly*).	DY Y DK N DN
15.	The plant has an 'accidents at work' information system (*e.g. a mannequin with locations of injuries, information board, newsletter*).	DY Y DK N DN
16.	In my firm, safe work performance by employees is promoted (*e.g. by being taken into account in the employee assessment process*).	DY Y DK N DN
17.	From time to time, my superior happens not to follow the safety rules.	DY Y DK N DN

Copyright © Marta Znajmiecka – Sikora
The use of the tool requires the written consent of the author

18.	The number of tasks I have to perform on a daily basis causes me to have to work at a very fast pace.	DY	Y	DK	N	DN
19.	My superior gives me feedback on my work performance, bringing attention to the fact that I must work safely.	DY	Y	DK	N	DN
20.	My superior's conduct sets an example for me in terms of safety.	DY	Y	DK	N	DN
21.	My superiors actively participate in safety promotion actions organised by the firm.	DY	Y	DK	N	DN
22.	Each and every accident that has happened in our plant is discussed by the superior during information meetings.	DY	Y	DK	N	DN
23.	The plant maintains a logbook of potentially dangerous incidents (near-accidents).	DY	Y	DK	N	DN
24.	The logbook of potentially dangerous incidents (near-accidents) is used, for example, to advise employees of the risks and prophylactic measures taken.	DY	Y	DK	N	DN
25.	My superiors 'turn a blind eye' on how work is performed – no matter whether or not it is done safely. What matters is timeliness and the required output.	DY	Y	DK	N	DN
26.	Particularly dangerous places are adequately marked.	DY	Y	DK	N	DN
27.	I usually work under time pressure.	DY	Y	DK	N	DN
28.	Our organisation has an action plan to improve occupational safety.	DY	Y	DK	N	DN
29.	The work I do is very strenuous for me.	DY	Y	DK	N	DN
30	My daily work schedule changes very often.	DY	Y	DK	N	DN
31.	I feel that my superior motivates me to work safely.	DY	Y	DK	N	DN
32.	After the entire day of work, I suffer from various muscular/backbone ailments (*e.g. corns, backbone pains*).	DY	Y	DK	N	DN
33.	The rules of moving around the plant are clearly specified.	DY	Y	DK	N	DN
34.	Machines and devices are repaired by qualified teams.	DY	Y	DK	N	DN
35.	Control elements of machines are well visible and marked.	DY	Y	DK	N	DN
36.	I have been advised of the occupational risk assessment at my workstation.	DY	Y	DK	N	DN
37.	I have a good relationship with my superior and I know I can rely on him/her in case of an emergency.	DY	Y	DK	N	DN
38.	When doing a dangerous job, I know I can trust my colleagues with whom I am fulfilling a dangerous task.	DY	Y	DK	N	DN
39.	I feel well-informed about protection against my occupational risks.	DY	Y	DK	N	DN
40.	I believe I am well-trained in providing first aid and if an accident were to happen, I would know what to do.	DY	Y	DK	N	DN
41.	I know the employee and the employer's duties in respect of safety.	DY	Y	DK	N	DN
42.	Upon change of my workstation – prior to starting work at a new workstation I am given the workstation instruction.	DY	Y	DK	N	DN
43.	I have a good relationship with my colleagues in the workplace.	DY	Y	DK	N	DN
44.	In our organisation safety is a priority, the process of improving safety is continuous.	DY	Y	DK	N	DN
45.	Communication within the team is difficult, there is no knowing who you can trust.	DY	Y	DK	N	DN
46.	Our organisation continuously takes various measures to improve work safety.	DY	Y	DK	N	DN
47.	In our organisation, safety issues are only discussed when an accident or inspection takes place.	DY	Y	DK	N	DN
48.	Our firm conducts practical exercises (*e.g. evacuation exercise, accident simulation*).	DY	Y	DK	N	DN
49.	When working in a team, I know I can trust the other team members.	DY	Y	DK	N	DN
50.	When doing work I know very well, I happen to flout the OSH rules from time to time.	DY	Y	DK	N	DN

Please check if you have answered all the questions!

Copyright © Marta Znajmiecka – Sikora
The use of the tool requires the written consent of the author
